# Intrathecal Baclofen in Hereditary Spastic Paraparesis

**DOI:** 10.3389/fneur.2019.00901

**Published:** 2019-08-23

**Authors:** Elke Pucks-Faes, Judith Dobesberger, Gabriel Hitzenberger, Heinrich Matzak, Andreas Mayr, Elena Fava, Eleonora Genelin, Leopold Saltuari

**Affiliations:** ^1^Department of Neurology, Hochzirl Hospital, Zirl, Austria; ^2^Department of Neurology, Paracelsus Medical University, Salzburg, Austria; ^3^Research Unit for Neurorehabilitation, Bolzano, Italy

**Keywords:** intrathecal baclofen, sporadic spastic paraparesis, hereditary spastic paraparesis, spasticity, device implantation

## Abstract

**Introduction:** Treatment with intrathecal baclofen (ITB) is a therapeutic option in the management of severe spasticity in patients with hereditary spastic paraparesis (HSP). However, information on the impact of ITB on the natural course of disease, especially the effect of ITB on functional parameters over time is limited.

**Materials and Methods:** We evaluated seven patients with HSP retrospectively who were treated with an ITB device. The following parameters were measured before *(pre-implantation)* and after implantation *(post-implantation)* of the ITB device at steady state dosage of ITB and annually until last follow-up: modified Ashworth Scale, Reflex Scale, modified Rankin Scale, and Rivermead Mobility Index. The ITB dosages were assessed after reaching steady state as well as annually until last follow-up.

**Results:** The ITB device was implanted 13 ± 6 (range 9–16) years after diagnosis of HSP on average. Severe spasticity was controlled in all patients by a mean baclofen dosage of 188 ± 60 (range 145–230) μg per day at steady state post-implantation. The modified Ashworth Scale improved significantly from 3 (interquartile range [IQR] 3–3.25) to 1 (IQR 1–1.25; *p* = 0.046), as did the Reflex Scale from 5 (IQR 4.75–5) to 3 (IQR 2.75–3; *p* = 0.046) at steady state dosage of ITB. The modified Rankin Scale improved from 2 (IQR 2–2) to 1 (IQR 1–1.5; *p* = 0.083) and the Rivermead Mobility Index remained 14 (IQR 13.5–14 pre-implantation, IQR 14–14 post-implantation; *p* = 0.18). Post-implantation, spasticity improved for 2–3 years, followed by a stable phase of ambulatory and other mobility functions for 4–5 years. Thereafter, the maintenance or progressive loss of mobility depended on individual courses of the disease. No ITB-related severe side effects occurred.

**Discussion:** Our data further support the role of ITB in the treatment of severe spasticity in patients with deteriorated walking performance suffering HSP. ITB therapy may initially improve spasticity and stabilize mobility functions for the first 6–8 years in patients with HSP.

## Introduction

Hereditary spastic paraparesis (HSP) constitutes a group of neurodegenerative disorders with heterogeneous genetic and clinical features characterized by a progressive spastic paraparesis and weakness of the lower extremities as the predominant symptoms. Depending on the presence of additional symptoms, such as cognitive impairment, epilepsy, cerebellar symptoms, or polyneuropathy, it can be divided into a pure (uncomplicated) and a complicated form. The course with initial symptoms as well as progression rate is variable in both, familial and sporadic cases of HSP (1–5).

So far, only symptomatic treatment of HSP is available with oral anti-spastic medication as first line medical treatment to reduce muscle spasticity this being the most disabling clinical symptom. In patients with severe spasticity, especially in those non-responding to oral antispastic medication or when the oral medication has limiting side effects, ITB is a potential therapeutic option. The use of ITB in spasticity of spinal origin was first described in 1984 (6) and its efficacy proven in several studies (7, 8).

The review of the literature revealed only few reports on the use of ITB in patients with HSP, these being mainly retrospective case reports or small patient series (9–16). An intrathecal bolus of baclofen improved gait control in a patient with pure HSP compared to healthy subjects (17). Lambrecq et al. reported four patients with HSP who received an ITB device after assessment of spasticity, muscular strength and walking performance (15). A high degree of satisfaction was noted in patients who were implanted at an early stage of the disease with stable functional results during the first 5 years of ITB treatment. The largest series on ITB for symptomatic treatment of HSP is prospective and reported on 14 patients (18). All patients had a significant reduction in lower limb spasticity and walking abilities. Complications were catheter fractures in 2/14 patients with consecutive surgical interventions.

However, information on the effect of ITB in patients with HSP is still limited, especially on the association between ITB and the natural course of the disease. Specific information about the ITB dosage requirement after ITB device implantation over time is also still lacking. The aims of this study were to investigate the long term effect of ITB on functional parameters including safety and adherence as well as dosages of chronic ITB treatment in patients with HSP.

## Materials and Methods

We identified 11 patients with HSP retrospectively who were treated with an ITB device implantation at the Department of Neurology, Hochzirl Hospital, Zirl, Austria, between 01.01.1990 and 30.11.2018. Seven out of 11 patients entered the final analysis, 4 were sporadic and 3 familial cases of HSP. Four patients were excluded as they were only admitted once to our department for ITB testing procedure and ITB device implantation, but followed-up at their local district hospital as our hospital is also a reference center for neurorehabilitation.

The decision to implant an ITB device was made in an interdisciplinary setting after an ITB testing phase (three patients baclofen bolus trial, four patients continuous baclofen trial). An ITB device was implanted, when the ITB test trial resulted in a decrease by at least 1 point of the modified Ashworth scale, in preserved muscular strength and improved gait function. All surgical interventions regarding the ITB device (implantation of ITB device, revision surgery due to end of battery life, revision surgery after device-related complication) were performed at the Department of Neurosurgery, Medical University Innsbruck, Austria.

We collected the following data in all included patients: demographic information (gender, age), sporadic vs. familial presentation, pure vs. complex form, time interval from diagnosis of HSP to implantation of the ITB device, device-related complications, ITB-related complications, replacement surgeries due to end of battery life or due to revision after a device-related complication and duration of follow-up. Several parameters were measured to evaluate the effect of ITB before implantation of the ITB device *(pre-implantation)* as well as after ITB device implantation *(post-implantation)* at steady state dosage of ITB (32 days post-implantation on average), and annually until last consultation of follow-up: the modified Ashworth Scale of the most affected lower limb (a 6-point scale for assessing spasticity; see Table 1) (19), the Reflex Scale (0–5 point scale of deep-tendon reflexes at patella and achilles; Table 1) (10), the modified Rankin Scale (a 6-point scale indicating general functioning in activities of daily life; Table 1) (20) and the Rivermead Mobility Index (21). The Rivermead Mobility Index measures body mobility with a 0–15 point scoring system and concentrates on disabilities in gait and transfer. It comprises 14 questions and one direct observation and covers a range of activities from turning over in bed to running. Based on three broad stages of response to ITB therapy as described below, we assessed the median scores of the four functional parameters of stage 1 (comprising first 2.5 years of ITB treatment), stage 2 (comprising following 4.5 years of ITB treatment), the first 3 years of stage 3 (comprising the 7th to the 10th year of ITB therapy), and following 10 years of stage 3 (comprising the 11th to the 20th year of ITB treatment. Only patients with sufficient data entered the particular analyses. The duration of follow-up was defined as period from implantation of the ITB device until date of last consultation. Four patients had a long follow-up period of at least 19 years (patients 4, 5, 6, and 7), in three of them, data were fully available only during the last 9 years of follow-up on average (patient 5, 6, and 7). In these patients, single data were applicable before, f.e. date of implantation of the ITB therapy, ambulatory/non-ambulatory status, modified Ashworth Scale pre-implantation etc. derived from patient records. Therefore, f.e. the patients 1, 2, 3, and 4 entered the particular analysis of the functional scores pre-implantation compared to post-implantation (but patients 5, 6, and 7 excluded due to incomplete data). A detailed gait analysis with video documentation was performed repeatedly in 4/7 patients before and/or after ITB device implantation with difficulties in finding the individual baclofen dosage requirement. Here, the Rivermead Visual Gait Assessment (RVGA) was used to measure relevant aspects of the quality of gait during the stance and swing phase such as position of the trunk, movement of pelvis, knee flexion, and extension or ankle plantar/dorsiflexion (22).

**Table 1:** Functional measurements for assessing spasticity (modified Ashworh Scale), reflexes (Reflex Scale), and general functioning in activities of daily life (modified Rankin Scale).

**Modified Ashworth Scale (19)**	
0	No increase in tone
1	Slight increase in tone, manifested by a catch and release or by minimal resistance at the end of the range of motion in flexion or extension
1+	Slight increase in tone, manifested by a catch, followed by minimal resistance throughout the remainder (less than half) of the range of motion
2	More marked increase in tone through most of the range of motion, but affected part easily moved
3	Considerable increase in tone; passive movement difficult
4	Affected part rigid in flexion or extension
**Reflex Scale (10)**	
0	Reflexes absent
1	Hyporeflexia
2	Normal
3	Mild hyperreflexia
4	Three or four beats clonus only
5	Clonus
**Modified Rankin Scale (20)**	
0	No symptoms.
1	No significant disability; able to carry out all usual activities, despite some symptoms.
2	Slight disability; able to look after own affairs without assistance, but unable to carry out all previous activities.
3	Moderate disability; requires some help, but able to walk unassisted.
4	Moderately severe disability; unable to attend to own bodily needs without assistance, and unable to walk unassisted.
5	Severe disability; requires constant nursing care and attention, bedridden, incontinent.

The course of the ITB dosages were assessed post-implantation firstly after reaching steady state of ITB defined as ITB dosage at discharge from hospital after ITB device implantation and in succeeding consultations annually until the last consultation of follow-up. Additional oral and local antispastic medication after ITB device implantation was also evaluated.

### Statistical Analysis

Data were summarized in cross tables. The median and interquartile ranges [IQRs] were calculated for ordinal variables. The Wilcoxon signed-rank test for paired observations was used to test for changes of these parameters before compared to steady state concentration of the ITB treatment. The means, standard deviation and ranges were calculated for metric variables. Statistical significance was set at a 2-tailed value of *p* < 0.05. We used SPSS, version 24.0 (IBM Corp., Armonk, NY, USA) to analyse the data.

According to the Austrian law on research, retrospective observational studies do not require approval of the ethics committee.

## Results

**Figure 1:**
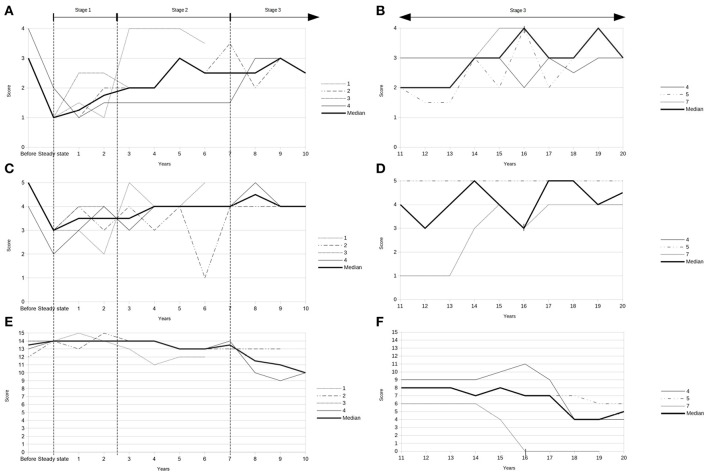
**(A)** Modified Ashworth Scale pre-implantation (“before”) and during first decade after implantation of the ITB device (data of patients 1, 2, 3, and 4). **(B)** Modified Ashworth Scale during second decade post-implantation (data of patients 4, 5, and 7). **(C)** Reflex Scale pre-implantation (“before”) and during first decade after implantation of the ITB device (data of patients 1, 2, 3, and 4). **(D)** Reflex Scale during second decade post-implantation (data of patients 4, 5, and 7). **(E)** Rivermead Mobility Index pre-implantation (“before”) and during first decade after implantation of the ITB device (data of patients 1, 2, 3, and 4). **(F)** Rivermead Mobility Index during second decade post-implantation (data of patients 4, 5, and 7). | = start of non-ambulatory status 16 years after ITB treatment (patient 7).

Demographic and clinical data of the seven patients treated with an ITB device for HSP are given in Table 2. The ITB devices were implanted between January 1990 and April 2015, which was 13 ± 6 (range 9–16) years after diagnosis of HSP on average. The implantation of the ITB device resulted in an improvement of clinical and functional parameters at steady state dosage of ITB compared to pre-implantation status (data of patient 1, 2, 3, and 4): the modified Ashworth Scale of the most affected lower limb decreased significantly from 3 (IQR 3–3.25) to 1 (IQR 1–1.25; *p* = 0.046), the Reflex Scale of the most affected lower limb improved significantly from 5 (IQR 4.75–5) to 3 (IQR 2.75–3; *p* = 0.046), the modified Rankin Scale decreased from 2 (IQR 2–2) to 1 (IQR 1–1.5; *p* = 0.083) and the Rivermead Mobility Index remained 14 (pre-implantation IQR 13.5–14, post-implantation IQR 14–14; *p* = 0.18). The course of the modified Ashworth Scale, the Reflex Scale and the Rivermead Mobility Index from pre-implantation over the first 10 years post-implantation as well as during the second decade post-implantation is given in Figure 1. We found three broad stages of response to the ITB treatment in patients with HSP: 1. In the first 2–3 years a significant reduction of spasticity as well as some tendency of improvement in mobility functions (stage 1) 2. In the following 4–5 years a rather stable phase of spasticity and functional capacity with no deterioration of gait and other mobility functions (stage 2) and 3. In the last two decades post-implantation either a slowly progressive (2/4 patients) or more progressive loss (2/4 patients) of ambulatory and other mobility functions in the last two decades post-implantation (stage 3). Table 3 displays the medians of the modified Ashworth Scale, the Reflex Scale, the modified Rankin Scale, and the Rivermead Mobility Index depending on the stage of ITB response. It reflects rather stable scores in the first 6–8 years of ITB treatment followed by an initially slow and then more pronounced deterioration of the scores during stage 3.

**Table 2:** Demographic and clinical data of 7 patients with hereditary spastic paraparesis treated with an intrathecal baclofen device.

**No**	**Age**	**Familial/ sporadic HSP**	**Pure/complex form of HSP**	**Time between diagnosis and device implantation (years)**	**Rivermead mobility Index pre-implantation**	**Device-related complications**	**Surgery due to end of battery life/complication**	**Follow-up (years)**
1	50-54	Familial^*^	Pure	16	14	Unknown catheter dysfunction	0/1	6.6
2	30-34	Familial	Pure	13	12	Catheter dislocation 2 x	1/2	9.3
3	10-14	Sporadic	Complex (cognitive impairment)	13	14	Liquor fistula	0/1	3.0
4	45-49	Sporadic	Complex (ataxia, dysarthria)	9	13	Catheter dislocation	2/1	19.0
5	45-49	Sporadic	Pure	15	Ambulatory^**^	None	2/0	18.4
6	40-44	Familial	Pure	10	Ambulatory^**^	Catheter dysfunction due to porosity	3/1	28.4
7	55-59	Sporadic	Pure	16	Ambulatory^**^	Infection with consecutive device explanation 2012	3/0	19.6^***^

*HSP, hereditary spastic paraparesis; m, man; No, Number; w, woman*;

* *mutation in SPG4*;

** *in patients 5, 6, and 7 Rivermead Mobility Index score pre-implantion not available, but “ambulatory” status according to patient records*;

*** *died 2014 from paralytic ileus*.

**Table 3:** Course of the modified Ashworth Scale, the Reflex Scale, the modified Rankin Scale, and the Rivermead Mobility Index depending on the stage of ITB response.

	Stage 1 (2–3 years)^*^	Stage 2 (4–5 years)^*^	Stage 3 (first 2-4 years)^*^	Stage 3 (second decade)^*^
Median modified Ashworth Scale (IQR)	1.5 (1, 2)	2 (1.5–3.5)	3 (2.25–3)	3 (2, 3)
Median Reflex Scale (IQR)	3 (3, 4)	4 (3, 4)	4 (4)	4 (4, 5)
Median modified Rankin Scale (IQR)	1 (1, 2)	2 (1, 2)	2 (1.5–2.5)	3 (3, 4)
Median Rivermead Mobility Index (IQR)	14 (14)	13 (13, 14)	13 (10-13)	7 (4-8)

* *stage 1, data of patients 1, 2, 3, 4; stage 2, data of patients 1, 2, 3, 4; stage 3 (first 2-4 years), data of patients 2, 4; stage 3 (second decade), data of patients 4, 5 7*.

**Figure 2:**
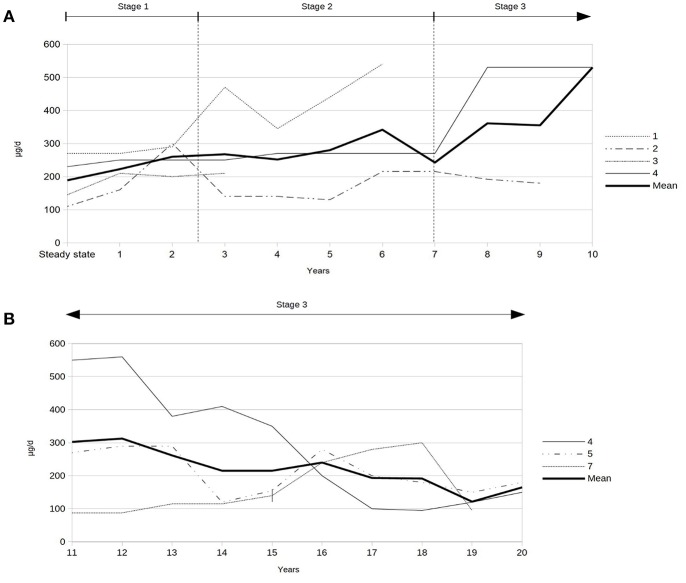
**(A)** Intrathecal baclofen dosages during first decade after implantation of the ITB device (data of patients 1, 2, 3, and 4). **(B)** Intrathecal baclofen dosages during second decade after implantation of the ITB device (data of patients 4, 5, and 7). | = start of non-ambulatory status 16 years after ITB treatment (patient 7).

Severe spasticity was controlled by a mean baclofen dosage of 188 ± 60 (range 145–230) μg per day at steady state post-implantation (data of patient 1, 2, 3, and 4). The mean ITB dosage at last follow-up of the seven patients was 231 ± 146 (range 95–540) μg per day. After 20 years of ITB treatment, the mean ITB dosage was 122 ± 28 (range 95–150) μg per day (patient 4, 5, and 7). A video-based gait analysis using the RVGA was carried out repeatedly in 4/7 patients which was helpful for fine-tuning of the individual ITB dosage requirement (three times pre-implantation and eight times post-implantation in patient 3, two times pre-implantation and five times post-implantation in patient 1, five times post-implantation in patient 4, one time pre-implantation and three times post-implantation in patient 2). The course of the ITB dosages post-implantation over the first 10 years, the second decade, respectively, is displayed in Figure 2. While the ITB dosages showed a slight increase during stage 1 on average, the dosages remained rather stable in phase 2, followed by a transient increase of the daily ITB dosages in the first years of stage 3 with subsequently decreasing ITB dosages. Co-administered oral antispastic medication could be stopped at steady state of ITB treatment in 3/4 patients (patient 2, 3, and 4), one of whom received botulinumtoxin B intermittently in addition during follow-up (patient 4). In the remaining patient (patient 1), oral antispastic therapy could be withdrawn 1 year after ITB device implantation.

All patients tolerated ITB treatment without any severe side effects. Device-related complications occurred in 5/7 patients (see Table 2). No ITB-related side effects occurred. The mean follow-up was 15.0 ± 8.9 (range 3.0–28.4 years). Two out of four patients with a long follow-up period of at least 19 years were still ambulatory with two walking aids at their last follow-up 19 years post-implantation (patients 4, 5). Of the remaining two patients, who were non-ambulatory at their last follow-up, one patient was ambulatory over 24 years (patient 6, follow-up 28 years) and the other patient was ambulatory over 16 years (patient 7, follow-up 19 years, then ITB device explanted as no longer clinical advantage compared to oral baclofen; patient died 2 years later from paralytic ileus).

## Discussion

We report our experience with the use of ITB in seven patients suffering HSP within an observation period of 29 years. ITB resulted in an improvement of spasticity for 2–3 years followed by a rather stable phase of 4–5 years on average both clinically and functionally. In the subsequent decade, the main targets of ITB therapy depended on the individual course of HSP with maintenance of mobility for as long as possible in still ambulatory patients or ease of care in non-ambulatory patients. Device-related complications occurred in the majority of patients (6/7; 86%), while no ITB-related severe side effects were reported.

So far, the majority of published papers on ITB in severe spasticity due to HSP comprise retrospective reports and small patient series (9–16). Some of these studies included patients with intractable spasticity of any etiology with only single or a few patients with HSP (9, 11). Ochs and co-workers published a prospective multi-center study on 28 patients with severe para- or tetraspasticity, of whom only one patient had the diagnosis of HSP (9). The patient had a considerably long disease duration of 29 years before implantation of the ITB device. In the study of Lambrecq et al. (15), the disease duration of 6 patients was 19 years on average, while the mean disease duration of our patients was 13 years. The shorter disease duration in our study cohort might be the reason for the comparatively high level of functioning suggesting a possible selection bias of the patients. An earlier referral of patients with otherwise intractable spasticity for ITB treatment in the course of the last 20–30 years might possibly explain the shorter disease duration in our study. However, we can only speculate on the correlation to a higher level of functioning, as in other papers, the disease duration can only be estimated [“leg stiffness since early childhood” (12), “at least a 5-year history of progressive disabling spasticity” (10), “progressive walking difficulties during the last 5 years” (16)] or is not given (13, 17, 18).

Meytaler and co-workers reported a significant improvement of muscle tone (2.04 points) and the reflex score (2.25 points) in three patients with HSP 3 months after implantation of the ITB device (10). Both the modified Ashworth Scale as well as the Reflex Scale also decreased significantly post-implantation in our study, although the assessments were conducted 1 month on average post-implantation. The ITB dosage for effective control of muscle tone and spasticity varied between 60 and 264 μg per day after 3 months in their study, whereas the effective ITB dosages at steady state varied less but were higher in our patient group (145–230 μg per day). The relatively high levels of daily baclofen dosages even in well-functioning subjects reflect the clinical practice in our center. We usually increase daily baclofen dosages gradually until the trunk stability is impaired, irrespective of the level of functioning. In our experience, functional capacity can be improved best with ITB therapy, when spasticity is decreased as far as possible with affecting the trunk stability as little as possible. For that purpose, functional tests in the vertical position are used, like the Rivermead Mobility Index or the Rivermead Visual Gait Assessment. These tests for gait analysis are more reliable to assess the best possible function capacity and to find individual baclofen dosage requirements compared to results of the modified Ashworth Scale and the Reflex Scale alone.

Margetis and co-workers published the largest series on ITB for symptomatic treatment of HSP in 14 patients with a prospective design (18). All patients had a significant reduction in lower limb spasticity according to the modified Ashworth scale, and a significant improvement of the walking abilities measured in a modified version of the functional walking scale of the Gillette Functional Assessment Questionnaire. The results of the modified Ashworth scale are comparable to our findings, whereby we measured walking abilities with the modified Rankin Scale and the Rivermead mobility index, which revealed non-significant improvements. The rate of complications was lower in the study of Margetis (14%; 2/14 catheter-related problems with consecutive surgical interventions) compared to our study (86%; 6/7 patients). This might be conceivably explained by the shorter follow-up period of 26 months on average in their patient group, while our study comprises a far longer follow-up period of nearly 10 years, thus detecting more complications according to the longer observational period.

Lambrecq and co-workers studied spasticity, muscle strength and walking ability in four patients with HSP and found “stable results of functional testing during the first 5 years of ITB-treatment” (15). But obviously, this was only derived from subjective descriptions of the patients as no assessment of functional parameters was performed. We analyzed follow-up data in seven patients with HSP and found three broad stages of response to ITB treatment according to the course of functional parameters: During stage 1, spasticity improved significantly in the first 2–3 years after implantation of the ITB device, while mobility function showed just some tendency to improve. During stage 2 of ITB treatment, both could be maintained rather stable over 4–5 years, clinical and functional parameters. In stage 3, clinical and functional parameters deteriorated more or less rapidly. The response to ITB was rather consistent during the first decade post-implantation, while it was dichotomous in the following two decades. Gait and other mobility functions could be either further maintained or deteriorated by trend in stage 3. This might be explained conceivably by the individual courses of the disease in patients with HSP. Accordingly, the main target of ITB therapy in patients with a less progressive course of HSP was preserving improved mobility functions whereas ease of care was the main goal of ITB therapy in patients with a clinical deterioration based on a more progressive course of disease.

ITB therapy in patients with HSP is challenging and often necessitates a compromise between decreased spasticity but preserved muscular strengthening. It demands an individualized approach respecting the grade of spasticity and muscular strengthening as most patients use their spasticity to compensate for muscle weakness.

Despite individual courses, the natural history of HSP is unremitting and at least slowly progressive in all patients. The results of our study suggest an improvement of spasticity during the first 2–3 years of ITB treatment, whereas both functional scores, the Rivermead Mobility Index even more than and the modified Rankin Scale, only show some tendency to improve during the first 2–3 years. In the following 4–5 years of ITB treatment, we found a rather stable phase of gait and other mobility functions including balance capacity. The gradual loss of balance capacity in the natural course of the disease is only weakly related to the increasing spasticity over time but correlates to the progressive muscular weakness affecting the trunk stability more likely. Scores for measuring spasticity as well as mobility functions start to deteriorate progressively after 6–8 years of ITB therapy, after about 20 years of disease duration, respectively. Thus, the continuous process of the disease might be postponed with ITB treatment for about 6–8 years.

To our knowledge, this is the first study demonstrating the impact of ITB on the course of HSP over a prolonged period of time with three subsequent stages after implantation of an ITB device correlated to ITB dosages. The initial improvement of spasticity correlates to slightly increasing ITB dosages during stage 1. The following clinically and functionally rather stable phase correlates to also rather stable ITB dosages during stage 2. After about 6–8 years of ITB treatment, both courses, the less or more pronounced clinical deterioration of function correlate to an initial increase of the ITB dosages followed by decreasing dosages (stage 3). After 20 years of ITB treatment, the daily ITB dosage accounts for roughly half of the ITB dosage at steady state post-implantation. The decrease of the ITB dosages during stage 3 might suggest a “pseudo-tolerance” to ITB, but based on our data, the response to ITB seems to be influenced by different progressive courses of HSP. Furthermore, the decrease of the ITB dosage starting after 10 years of ITB treatment reflects most likely the attempt to preserve any mobility function with respect to the natural course of the disease.

The main limitations are firstly the retrospective assessment of the data and secondly the small number of included patients from our single center. Furthermore, the results are limited as the study lacks a control group. Moreover, the patients have different durations of follow-up and in three patients with a long follow-up period of at least 19 years, follow-up data were fully available only during the last 9 years on average.

However, our data might further elucidate benefits as well as limitations of the ITB therapy in patients with HSP. Treatment with ITB might postpone the progressive course of the disease resulting in a transient clinical improvement and then maintenance of function for nearly a decade. Thereafter, the response to ITB with regard to mobility function seems to be limited by the individual natural course of the disease and thus requires different goals of ITB treatment. The findings of the present study might serve as recommendations for physicians considering an ITB treatment in patients with HSP. They might also be helpful for counseling the patients on the benefits with an improvement of spasticity and stabilization of mobility functions for at least 6 years and as well as risks and limitations of the ITB therapy based on the individual progression of the disease. A large multicenter study is necessary to confirm the findings of this study and, thus, further investigate the role of ITB in the management of severe spasticity in HSP.

## Data Availability

The datasets generated for this study are available on request to the corresponding author.

## Ethics Statement

According to Austrian law on research, retrospective observational studies do not need approval of the ethics committee.

## Author Contributions

EP-F worked substantially on the preparation of the manuscript with regard to the conception, analysis, and interpretation of the data at every stage from the beginning until the submission of the paper. JD contributed substantially on the preparation and correction of the manuscript. GH contributed substantially in the data acquisition of the patient records and conception of the manuscript and performed the statistical calculations of the paper and corrected all parts of the manuscript. HM contributed substantially in the data acquisition of the patient records as well as corrected all parts of the manuscript. AM contributed substantially in the data acquisition of patients with a detailed video-based gait-analysis and corrected all parts of the manuscript. EF contributed substantially in the data acquisition of the patient records, also worked on the section Methods and Materials and corrected all parts of the manuscript. EG contributed substantially in the data acquisition of the patient records, worked also on the draft of the section Methods and Materials and corrected all parts of the manuscript. LS contributed substantially in all parts of the manuscript at each time and stage of the paper from conception, analysis, and interpretation of the data until submission. All authors provided approval for publication of the content and agreed to be accountable for all aspects of the work in ensuring that questions related to the accuracy or integrity of any part of the work are appropriately investigated and resolved.

### Conflict of Interest Statement

The authors declare that the research was conducted in the absence of any commercial or financial relationships that could be construed as a potential conflict of interest.
